# NaCl substrates for high temperature processing and transfer of ultrathin materials

**DOI:** 10.1038/s41598-020-64313-9

**Published:** 2020-04-29

**Authors:** Christina Graham, Miriam Marchena Martin Frances, Rinu Abraham Maniyara, Yugeng Wen, Prantik Mazumder, Valerio Pruneri

**Affiliations:** 1 ICFO- Institut de Ciències Fotòniques, The Barcelona Institute of Science and Technology, 08860 Castelldefels, Barcelona Spain; 2 Corning Research and Development Corporation, Sullivan Park, Corning, New York, NY 14831 United States of America; 3 ICREA- Institució Catalana de Recerca i Estudis Avançats, 08010 Barcelona, Spain

**Keywords:** Synthesis and processing, Two-dimensional materials

## Abstract

Ultrathin materials often require high temperatures for growth and processing, which cannot be withstood by the substrate underneath. For example, polymers are widely used as a supporting layer but unfortunately have low strain-point temperatures. This is the case of polyethylene terephthalate (PET) which has glass transition and melting temperatures of 76 and 250 °C, respectively. In this paper we propose to use polished salt, a material that can withstand high temperatures during fabrication and, at the same time, can be sacrificed during the transfer onto the final substrates. More specifically, we demonstrate thermal dewetting of Au ultrathin metal films and growth of MoS_2_ on NaCl at 750 and 650 °C, respectively, and subsequent transfer onto PET films, after which the salt is easily dissolved by water. We believe that the proposed technique can be extended to fabrication of other ultrathin materials, e.g. graphene, as well as final substrates for a wide range of applications, including flexible electronic and optoelectronic devices.

## Introduction

The study of thin materials is an ever-growing interdisciplinary topic spanning the fields of electronics, engineering, chemistry, physics and materials science not to mention the heightened commercial interest. The field covers materials with thicknesses at the nanometer scale and recently, down to the atom scale, which are known as two-dimensional (2D) materials^1^. The great interest of such materials stems from their remarkable properties arising from the quantum confinement, electrical transport and optical effects that emerge when scaled to very low thicknesses, which is of particular interest for optoelectronic applications. Over the last decade or so, the potential of thin materials has been extrapolated down to 2D materials, where thicknesses have been reduced to the atomic scale, with graphene being the first one to be isolated in 2004 by A. Geim and K. Novoselov^2^. Since then, the 2D materials portfolio has been expanded to, for example, hexagonal boron nitride (hBN)^3^, transition metal dichalcogenides (TMDs)^4–7^, layered double hydroxides (LDHs)^8^, and also different 2D heterostructures resulting from the combination of previous materials in a vertical stack due to van der Waals forces^9^. Of the aforementioned materials, TMDs have been the focus of intense research owing to their non-zero tunable band gap, optical transparency and mechanical flexibility^10–13^. Of particular interest is MoS_2_ which undergoes a indirect to direct band gap transition when thinned to a monolayer^14^. Furthermore, it is thermally stable up to 1100 °C, with a high bulk carrier mobility (200–500 cm^2^V^−1^s^−1^)^15^, and large on/off ratio (10^8^)^16^. Due to the aforementioned properties, MoS_2_ has already found application in FETs^11,16^, catalysis^17–19^, energy storage^20^ and sensors^21^ with recent work demonstrating potential for flexible electronics applications^11,13,22,23^.

Another material of interest in optoelectronics is ultrathin metal films (UTMFs), with thicknesses below 10 nanometers, which have been widely studied due to their interesting electrical transport and optical properties (e.g. high transmittance, good conductivity and low sheet resistance), and also their easy deposition on a wide variety of substrates (e.g. rigid and flexible)^24,25^. Furthermore, exposing UTMFs to high temperature drives a phenomena called “dewetting”, whereby the metal film spontaneously retracts to form isolated nano-particles^26^. The easy fabrication of these structures widens the number of applications making them suitable for large scale applications, such as masks to fabricate nanostructured surfaces for antireflective and antiglare surfaces^27^ and structural coloring^28^.

However, the implementation of materials such as MoS_2_ and dewetted UTMFs in flexible devices is challenging due to the high temperatures needed for their fabrication, which are not compatible with some low cost and flexible polymeric substrates, such as PET. Due to this limitation, these materials are typically grown on SiO_2_ or sapphire, which can withstand high temperatures, after which they can be transferred onto a flexible substrate *via* a polymethyl-methacrylate (PMMA)-assisted wet etching step. In this procedure, the polymer is used as a support and then, a chemical etchant is used to remove the substrate releasing the PMMA/film. However, the etchant, usually hydrogen fluoride (HF) or a strong base (NaOH or KOH), can damage the film^29^. Therefore, extending the applications of MoS_2_, dewetted UTMFs and related devices requires the development of a method to transfer high thermal processing materials onto polymeric substrates.

The use of water-soluble sacrificial materials is common practice in the growth and transfer of materials onto arbitrary substrates. The sacrificial material can be utilized as either the growth substrate or as an intermediate sacrificial film. For example, NaCl has been used as a sacrificial substrate to obtain TEM diffractograms and bright field images of sputter coated ZnS and GaAs^30^. The growth of ZnO^31^, magnetic materials^32^, metallic films^33,34^ and the fabrication of micromachined nanostructures has also been demonstrated^35^. Meanwhile, sacrificial NaCl films have found use in transfer printing, nanotexturing^36,37^ and for the fabrication of metallic nanostructures for transparent flexible electrodes^38^. With reference to the work contained in this paper, MoS_2_ growth on NaCl has been previously demonstrated by a method of sequential deposition of Mo and S layers by sputtering and thermal evaporation, respectively^39^. The MoS_2_ films were synthesized by a solid state reaction, induced by annealing, between the Mo and S constituents in thin film form. By this method the authors demonstrated the fabrication of textured and photoactive MoS_2_ on NaCl.

In this work, we demonstrate the use of sacrificial NaCl substrates for the transfer of high thermal processing materials. As prototypical examples we investigate gold dewetted nanoparticles (Au DNPs) and MoS_2_ onto PET substrates that cannot withstand their fabrication temperatures, 750 °C and 650 °C respectively. The transfer of the deposited material onto the target substrate consists in a fast detachment when water penetrates in between, a process that takes only several minutes. For both structures, we demonstrate the preservation of the film quality after the transfer and the non-alteration of electrical and optical properties. Thus, the proposed technique allows an etching-free, fast and easy transfer, which could be extended to other materials, such as graphene, requiring high temperature processing.

## Methods

### NaCl substrate preparation

NaCl substrates from International Crystal Laboratories with a size of 1 × 1 inches and of 5 mm thickness were used for the Au DNPs, meanwhile for MoS_2_, the substrate was diced to 0.5 × 0.5 inches in order to fit the furnace dimension. PET films of 125 µm thickness from Goodfellow Inc. were used as the flexible target substrate. All substrates were sonicated in conventional organic solvents for 10 minutes and dried with a N_2_ gun.

### Au DNPs fabrication

Gold (Au) thin films of 7 nm thickness were deposited onto NaCl substrates using a Lesker thermal evaporator with a deposition rate of 1 Å/s. The continuous films were subsequently dewetted by a rapid thermal annealing (RTP) at 750 °C for 90 seconds. A thorough description of the dewetting procedure can be found in a previous ref. ^26^. Note that the resulting nanoparticles have a nanocap geometrical shape^28^.

### MoS_2_ growth on NaCl substrate

**Figure 1:**
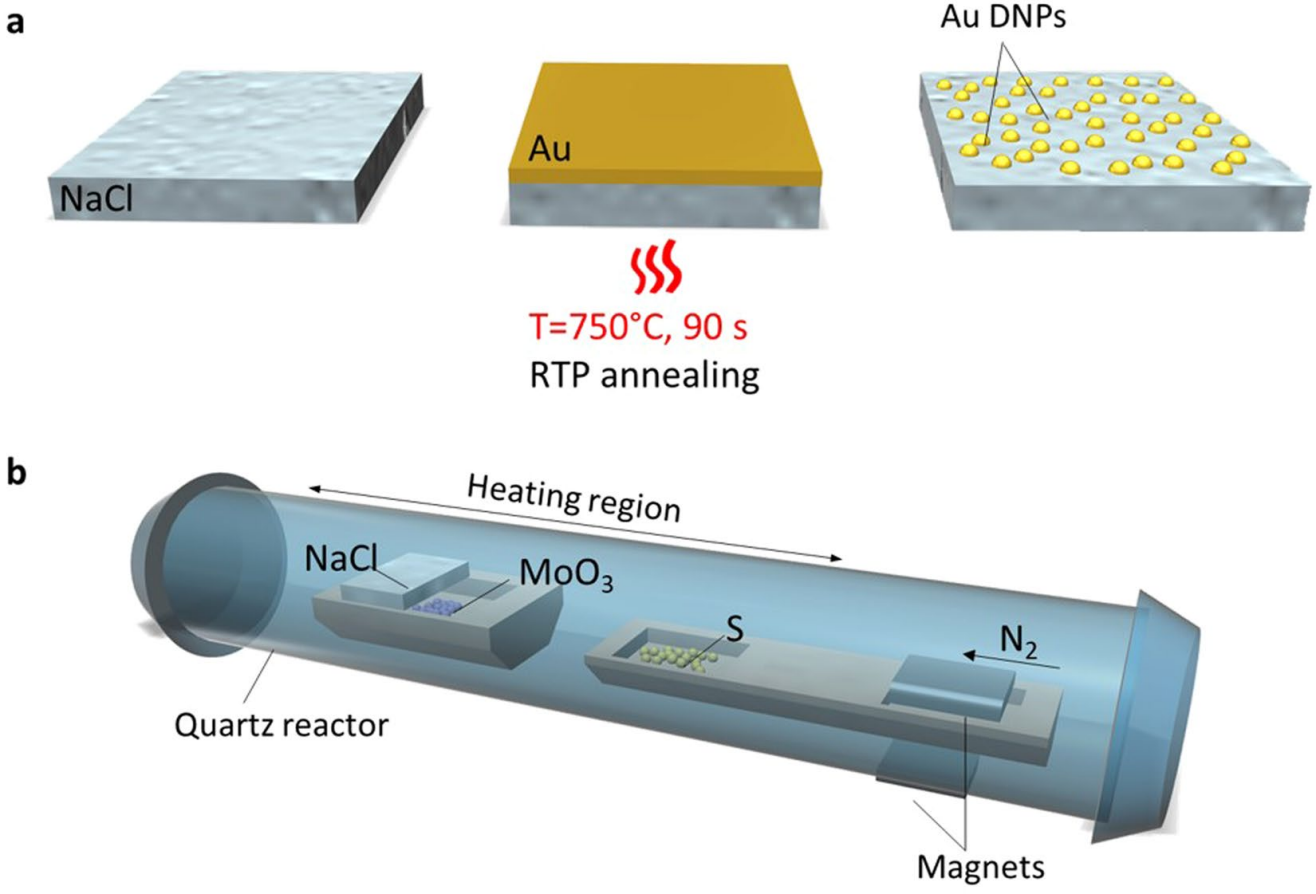
(**a**) Fabrication of Au dewetted nano-particles (nano-caps) on NaCl by heating a sputtered Au layer of 7 nm thickness at 750 °C for 90 seconds. (**b**) CVD growth of MoS_2_ on NaCl substrates due to the reaction of MoO_3_ with S powder, with the latter being introduced at 650 °C using two magnets.

The growth of MoS_2_ was performed using a typical chemical vapour deposition (CVD) process illustrated in Fig. 1. The NaCl substrate was mounted facing down above a ceramic boat containing 6 milligrams of MoO_3_ precursor (Sigma-Aldrich, 99.97% purity) and then loaded into the CVD furnace (MTI GSL-1100X-NT-LD). Another boat containing 300 milligrams of sulphur (Sigma Aldrich, 99.98% purity) and a magnet was placed upstream, 18 centimetres from the boat of MoO_3_, in order to control the rate of sublimation. The furnace was firstly heated to 300 °C with a rate of 20 °C min^−1^ after which the rate was reduced to 10 °C min^−1^ to prevent overshooting of the target temperature. Upon reaching a growth temperature of 650 °C, an external magnet was used to push the sulphur containing boat into the reaction zone where the temperature was held at 650 °C for 5 minutes. During the process, nitrogen (99.999% purity) was used as the carrier gas, with a flow rate of 50 standard-state cubic centimeter per minute (sccm). After 5 minutes, the furnace was left to cool down slowly. Atmospheric pressure was maintained throughout the experiment.

### Characterisation techniques

SEM images were obtained using a FEI-Scanning Electron Microscopy (FE-SEM, FEI Inspect F). Optical characterisation comprised of UV − vis−NIR spectrophotometric measurements (PerkinElmer Lambda 950). For MoS_2_ (before and after performing the transfer), micro-Raman analysis (InVia Renishaw, 532 nm laser excitation and 50X lens) was performed.

## Results and Discussion

### Development of the transfer procedure

**Figure 2:**
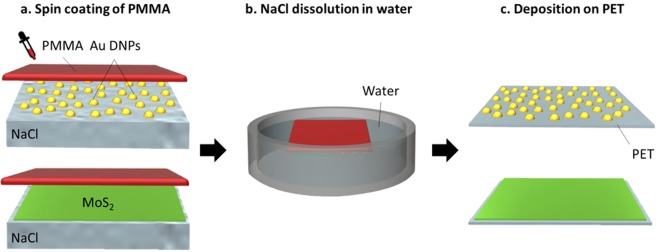
Transfer of Au DNPs and MoS_2_ to PET using PMMA as an intermediate layer: (**a**) Spin-coating of PMMA at 4000 rpm for 1 minute; (**b**) Partial dissolution of NaCl substrate in water to afford the separation of the growth material covered with PMMA; (**c**) PMMA with Au DNPs and MoS_2_ is located on top of the PET after which the PMMA is removed by immersion in acetone and isopropyl alcohol for 10 minutes each.

A polymer assisted wet transfer method was used to transfer the MoS_2_ film and the Au DNPs onto a flexible substrate. Figure 2 illustrates the common transfer process using a PMMA as an intermediate layer. Firstly, a PMMA film was spin-coated at 4000 rpm for one minute onto the sample surface as a support to avoid the disaggregation of both growth materials, MoS_2_ and Au DNPs respectively, during the transfer.

The PMMA layer, with the adhered growth material, is afforded by water intercalation in between the growth material and substrate due to the high solubility of NaCl in water. The substrate is first located on the base of the beaker after which deionised water is slowly added. Once that the water level achieves the top surface of the substrate, the release of the growth material occurs gently without inducing cracks or wrinkles. After that, the growth material remains floating on the water surface and it is then located onto the PET substrate. The process was performed at room temperature and was complete within minutes for the samples of 1 × 1 inch size. After locating the growth material on top of the PET, the PMMA is removed by immersion in acetone and isopropyl alcohol for 10 minutes each. In case of removing PMMA residues, cleaning could be improved by a low power oxygen plasma treatment. Thus, our method guarantees easy, fast transfer and, given the 5 mm thickness of the NaCl substrate, there is the potential to reuse the growth substrate by performing a post-transfer surface conditioning procedure. The supplementary Figure S1 provides AFM pictures of the NaCl substrate as received after cleaning, immediately after MoS_2_ transfer, and following a rapid thermal annealing surface conditioning treatment at 750 °C for 135 seconds, where it can be observed that the surface roughness is markedly improved to approaching that of the as received substrate.

### Results for the transferred Au dewetted nano-particles and MoS_2_

**Figure 3:**
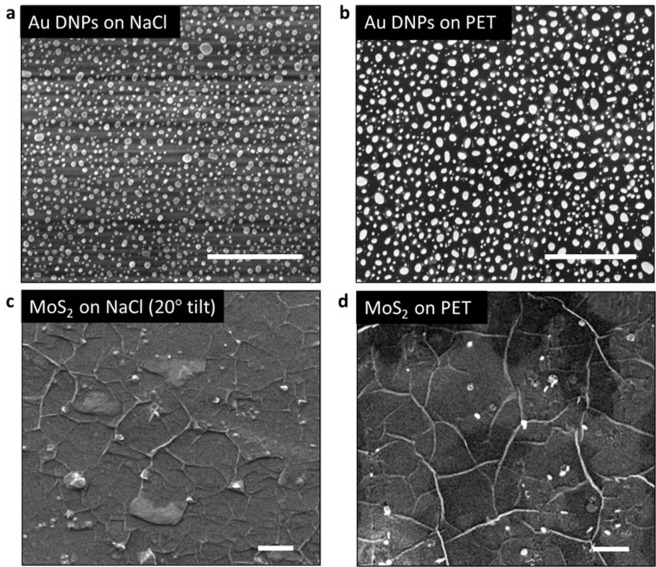
SEM images of (**a,c**) as-grown and (**b,d**) transferred Au DNPs and MoS_2_ respectively. Scale bar: 2 *μ*m. Note that the different contrast in the top images (**a,b**) is due to different substrate charging.

For the transfer to be successful, the coverage, morphology, thickness and quality of the material should be well preserved. In this section, we provide the corresponding characterisation (SEM, Raman and transmittance) before and after the transference of Au DNPs and MoS_2_ films. SEM imaging was performed to evaluate the morphology and uniformity of the Au DNPs and MoS_2_ films. Figure 3 shows a top view SEM of the Au DNPs and MoS_2_ before (Fig. 3a,c) and after (Fig. 3b,d) the transfer onto PET.

In both cases, the structure, coverage and morphology of the materials is preserved. In the case of Au DNPs, Fig. 3a,b confirm that the transfer did not significantly affect their size or distribution. Moreover, the supplementary Figure S2 provides an SEM comparison of Au DNPs on NaCl and fused silica where it is demonstrated that the morphology of the DNPs remains unchanged in each case, differing only in size due to the difference in interfacial energy between the Au and the substrate surfaces, respectively.

Regarding MoS_2_, the domains variation in shape and size as a function of precursor ratio has been well documented in the literature^40–42^ and can vary between hexagonal, triangular flakes and circular truncated and vertical stacks for more supersaturated conditions. In saturated conditions, as in our case where the Mo:S ratio is 1:50, the flakes have coalesced to form larger overlapping regions. This can be seen clearly in Fig. 3c,d, which show a continuous film with areas of overlapping domains.

**Figure 4:**
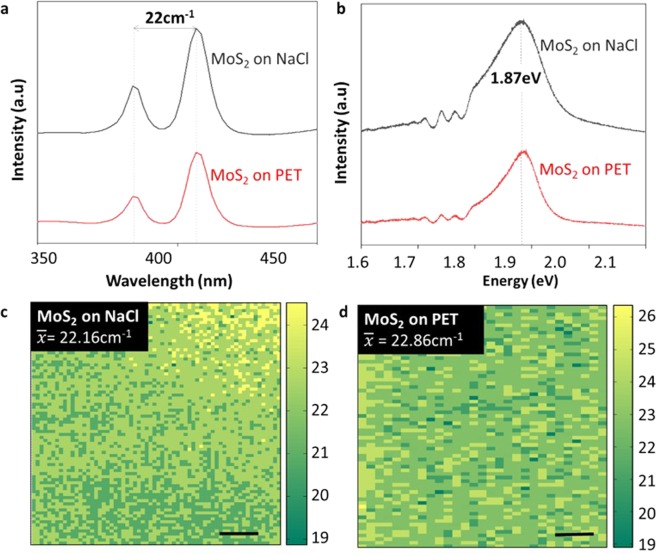
Single point (**a**) Raman and (**b**) photoluminescence spectra before and after transfer. Raman maps for MoS_2_ on (**c**) NaCl and (**d**) PET. Scale bar: 100 µm.

In addition to the visual quality of the films evaluated by SEM, Raman spectroscopy measurements were performed to identify the quality and layer thickness of the as-grown MoS_2_. Single point Raman spectra (Fig. 4a) displays the two signature peaks corresponding to the in-plane vibrations of the Mo and S atoms (E^1^_2g_) at ~385 cm^–1^ and the out of plane vibration of the S atoms (A_1g_) at ~407 cm^−1^ ^43^. Additionally, Raman maps (Fig. 4c,d) of 500 μm × 500 μm^2^ were obtained. For both the MoS_2_/NaCl and MoS_2_/PET maps, a uniform distribution and intensity was found with an average E^1^_2g_ − A_1g_ peak distance of 22 cm^−1^, therefore confirming that the MoS_2_ film was cleanly transferred with perfect preservation of the coverage, morphology, and thickness. The average peak separation of between 22 and 23 cm^−1^ is indicative of 2–3 layers of MoS_2_, which would agree with the SEM micrographs of Fig. 3c,d, which indicate several layer-overlapping domains. Moreover, single-point photoluminescence spectra (see Fig. 4b) shows the characteristic peak of A1 excitonic emission at 1.87eV^43^ for both the pristine and transferred MoS_2_ samples, again indicating a damage-free transfer with a perfect preservation of the sample.

**Figure 5:**
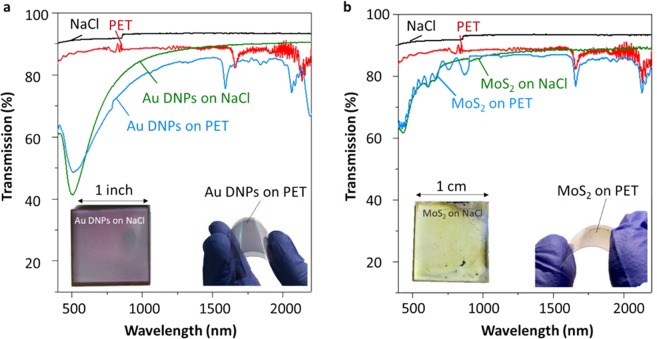
Transmission as a function of wavelength for (**a**) Au DNPs and (**b**) MoS_2_ on NaCl and PET. Inset shows photographs of Au DNPs and MoS_2_ on NaCl and PET substrate.

Finally, for applications where transparency is important, for example flexible displays and solar cells, the transmission of the films was measured in the range 400–2200 nm. The results are collected in Figs. 5a,b for Au DNPs and MoS_2_, respectively. All graphs include the transmittance of the bare NaCl (black line) and PET substrate (red line). Transmittance of the samples is preserved in both cases differing slightly due to the transmittance of the respective substrates. In the case of Au DNPs (see Fig. 5a), a transmission dip at 600 nm wavelength is present due to surface plasmon resonances^28^. Note that the reduced transmission is compared to Au DNPs on NaCl, which correlates to the reduced transparency of PET in the region 500–600 nm. The insets of Fig. 5a,b show the aspect of Au DNPs and MoS_2_, respectively, before and after transfer. The transferred films were completely removed from the growth substrate, leaving no visible PMMA residues and were deposited continuously, as confirmed by Raman mapping.

## Conclusions

This work demonstrates for the first time the growth of the following high thermal processing materials, Au DNPs and MoS_2,_ on NaCl substrates. Additionally, we show a new fast and easy way to transfer the growth materials to low strain point and flexible substrates, such as PET, whilst preserving the film quality. The technique is scalable, easy implemented and etching free. For case of fabricating metallic nanostructures, increased scalability only requires a larger sacrificial substrate when compared to existing methods such as nano-lithography which is always limited to less than micron size dimensions. Similarly, up-scaling the growth of MoS_2_ may be achieved with the use of a larger CVD chamber and consequent optimisation of the reactive compounds. In the future work, this technique could be extended to other ultrathin and 2D materials, such as graphene, offering path to the fabrication of a wide variety of high temperature processing 2D flexible devices.

## Supplementary information

Supplementary information.

## Data Availability

All data generated or analysed during this study are included in this published article. The datasets analysed during the current study are available from the corresponding author on reasonable request.
