# Supplementary material for: NaCl substrates for high temperature processing and transfer of ultrathin materials
Source: Sci Rep. 2020 Apr 29;10:7253. doi: 10.1038/s41598-020-64313-9 (PMC7190726; doi:10.1038/s41598-020-64313-9)
Supplement: Supplementary file 1 — Supplementary information. [file 41598_2020_64313_MOESM1_ESM.pdf]

Supplementary information

NaCl substrates for high temperature processing and transfer of ultrathin materials

Christina Graham[1], Miriam Marchena[1], Rinu Abraham. Maniyara[1], Yugeng Wen[1], Prantik Mazumder[2] & Valerio Pruneri[1, 3*]

[1] ICFO- Institut de Ciències Fotòniques, The Barcelona Institute of Science and Technology, 08860 Castelldefels (Barcelona), Spain.

[2] Corning Research and Development Corporation, Sullivan Park, Corning, New York, NY 14831, United States of America.

[3] ICREA- Institució Catalana de Recerca i Estudis Avançats, 08010, Barcelona, Spain.

Correspondence should be addressed to C.G (email: Christina.Graham@icfo.eu)

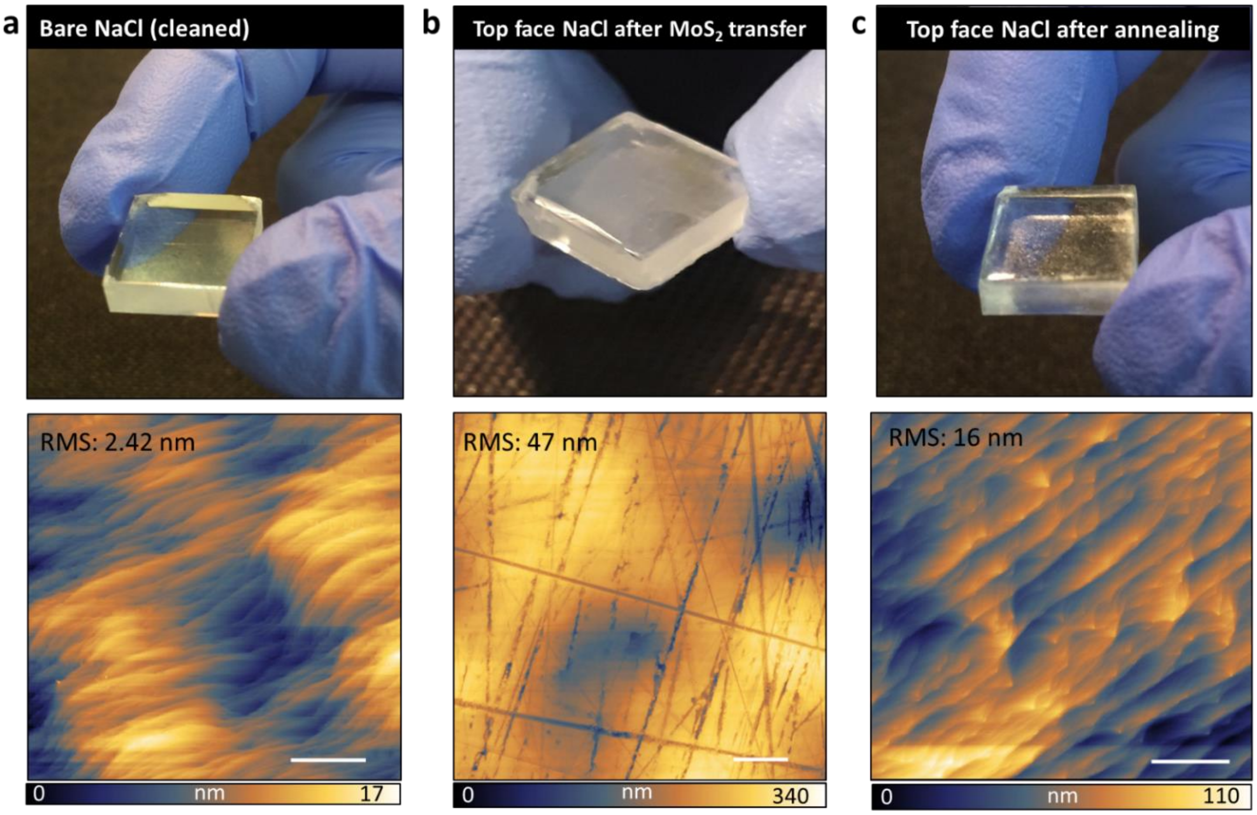

Figure S1 Photographs and AFM pictures of (a) NaCl substrate sonicated in conventional organic solvents for 10 minutes and dried with a N_2 gun , (b) after MoS_2 removal and (c) after post-transfer rapid thermal annealing at 750ºC for 135s. Scale bar: 5 μm.

Note that when utilising NaCl as a growth substrate, it is important to avoid exposure to air as much as possible because the NaCl is sensitive to humidity. If not kept under vacuum, the surface becomes rough due to the reaction with the humidity in the air. A rougher surface will lead to a poorer quality growth. This can be avoided by keeping the NaCl in vacuum and properly evacuating the air from the CVD chamber.

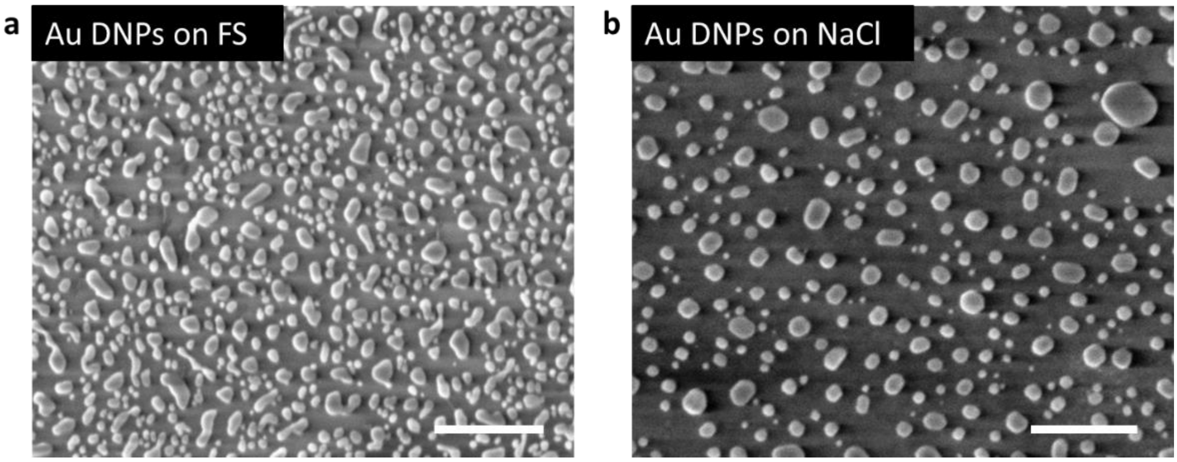

Figure S2: Gold (Au) thin films of 7 nm thickness were deposited onto Fused silica (FS) and NaCl substrates. The continuous films were subsequently dewetted by a rapid thermal annealing (RTP) at 750°C for 90 seconds. Scale bar = 500 nm.
